# Nanozymes as Emerging Therapeutics for Asthma: A Redox-Responsive and Immunomodulatory Strategy

**DOI:** 10.3390/biomedicines14051107

**Published:** 2026-05-14

**Authors:** Manar T. El-Morsy, Nadine M. Askar, Ali Emad Khurkhash, Nagm Al-Din Mahrous, Yusuf Ahmed Elberry, Mohamed Ramadan Sayed, Norhan Ashraf Ahmed, Rowayda A. Ahmed, Yehia S. Mohamed, Sinclair Steele, Ahmad Ahmeda, Rudaynah Mohamed, Doaa S. R. Khafaga

**Affiliations:** 1 Bio-Nanotechnology Department, Faculty of Nanotechnology, Cairo University, Giza 12613, Egypt; 14222023442600@pg.cu.edu.eg; 2 Biotechnology and Applications Program, Faculty of Science, Mansoura University, Mansoura 35516, Egypt; nadineaskar3@gmail.com; 3 Zoology and Chemistry Department, Faculty of Science, Al-Azhar University, Cairo 11884, Egypt; aliemad2036@gmail.com (A.E.K.); nagmmahrous74@gmail.com (N.A.-D.M.); 4 Faculty of Biotechnology, October University for Modern Sciences and Arts (MSA), 6th October City 12451, Egypt; yusuf.ahmed1@msa.edu.eg; 5 Faculty of Biotechnology, Nile University, Juhayna Square, 26th of July Corridor, El Sheikh Zayed, Giza 12588, Egypt; M.Ramadan2118@nu.edu.eg; 6 Biotechnology and Genetic Engineering Program, Faculty of Science, Helwan National University, Helwan 11795, Egypt; norhan.rashad.2004@gmail.com; 7 Pharmaceutical biotechnology Department, Faculty of Biotechnology, Badr University in Cairo, Badr City 11829, Egypt; rowayda.2022006508@buc.edu.eg; 8 Department of Pathological Sciences, College of Medicine, Ajman University, Al Jurf, Ajman P.O. Box 346, United Arab Emirates; s.steele@ajman.ac.ae; 9 Department of Microbiology and Immunology, Faculty of Pharmacy (Boys), Al-Azhar University, Cairo 11884, Egypt; 10 Department of Basic Medical Sciences, College of Medicine, University of Sharjah, Sharjah P.O. Box 27272, United Arab Emirates; aahmeda@sharjah.ac.ae; 11 College of Medicine, Ajman University, Al Jurf, Ajman P.O. Box 346, United Arab Emirates; 202510400@ajmanuni.ac.ae; 12 Department of Basic Medical Sciences, Health Sector, Galala University, New Galala City, Suez 43511, Egypt

**Keywords:** nanozymes, asthma, nanotechnology, immunotherapy, redox responsive

## Abstract

Asthma is a chronic, etiologically diverse lung disease that contributes to worldwide morbidity and healthcare burdens. Although bronchodilators and corticosteroids remain the cornerstones of asthma treatment, their long-term use is associated with significant side effects. Furthermore, steroid resistance in severe asthma emphasizes the need for alternative therapeutic approaches. Nanotechnology has emerged as a viable alternative to these standard approaches, allowing for targeted, prolonged, and precise drug delivery. Nanozymes, or synthetic nanomaterials that imitate natural enzyme functions, are gaining popularity among nanomedicine platforms due to their redox-regulating and immunomodulatory properties. This review provides a comprehensive overview of the present landscape of nanozyme-based treatments for asthma, with a focus on carbon-based nanozymes, while discussing MOF-derived and single-atom nanozymes in terms of their physicochemical properties and potential applicability to airway inflammatory diseases. Moreover, we look at current advancements in nanozyme-enabled drug delivery systems, their biocompatibility profiles, and potential strategies for designing nanozyme therapies according to asthma endotypes. These findings establish nanozymes as a transformational and therapeutically promising platform for next-generation asthma treatment.

## 1. Introduction

Asthma-like diseases have long existed in human history; Hippocrates (460–370 BC) described dyspnoea similar to modern asthma, and the Egyptians recognized similar respiratory problems. The name asthma, which comes from the Greek word aazein, meaning to breathe hard, became well-known in the eighteenth and nineteenth centuries as more scientific knowledge about the illness started to accumulate [1,2]. Asthma is a chronic and heterogeneous respiratory disease characterized by persistent airway inflammation, reversible airflow obstruction, bronchial hyperresponsiveness, and structural remodeling of the airways [3,4,5,6]. This persistent inflammation leads to common clinical symptoms such as wheezing, shortness of breath, coughing, and chest tightness, which vary in severity and frequency between individuals [7,8]. In recent years, according to the World Health Organization (WHO), asthma is a significant noncommunicable disease that causes disability and healthcare burdens worldwide. An estimated 262 million people globally suffer from asthma, with a significant proportion being children [3,9]. Although asthma treatments have noticeably improved over the past 15 years, allowing most patients to manage their symptoms effectively, the underlying causes of the disease remain poorly understood [3,6,10,11]. Both genetic and environmental factors contribute to asthma pathogenesis. Genetic predisposition affects allergen sensitivity, while environmental exposures trigger allergic inflammation [3,7]. Asthma’s pathological progression is driven by reduced lung function, allergic disorders, and respiratory infections, and is often linked to impaired lung activity beginning in the prenatal stage [8,12].

There are two main forms of asthma that have been identified: allergic and nonallergic asthma. Allergic asthma tends to begin in childhood and is associated with T helper 2 (Th2) cell responses, which are also seen in other allergic conditions such as atopic dermatitis or allergic rhinitis [8,13]. This form of asthma is induced by early life encounters with environmental allergens such as house dust mites (HDM), pollen, cockroaches, or animal dander, but can also be induced later in life when a new, e.g., occupational allergen, is encountered. Upon recognizing allergens, allergen-specific Th2 cells produce type 2 cytokines (interleukin [IL]-4, IL-5, IL-9, and IL-13) that lead to the accumulation of high numbers of eosinophils in the airway wall, mucus overproduction, and synthesis of immunoglobulin E (IgE) by allergen-specific B cells, which can be detected in the serum or through a positive skin-prick test [7,8,14,15].

On the other hand, non-allergic asthma (NAA), also known as intrinsic asthma, affects approximately 10–30% of asthmatic patients and typically presents later in life, with a female predominance [16]. Unlike allergic asthma, NAA is triggered by non-allergenic factors such as exercise, pollutants, hormonal changes, and viral infections. It is often associated with a low Th2 expression immune profile, where innate and adaptive immune responses, particularly involving Th17 cells, play a dominant role [17]. This phenotype is less responsive to inhaled corticosteroids and is characterized by elevated neutrophil activity and inflammatory mediators such as IL-17A, IL-8, TNF-α, and Leukotriene B4 (LTB4). Inflammatory dysregulation, including impaired resolution and IL-17–dependent pathways, contributes to persistent airway inflammation and exacerbations in NAA patients [5,7,15,18,19].

Current asthma treatments mainly rely on bronchodilators for symptom relief and corticosteroids for inflammation control [10]. However, long-term corticosteroid use can cause adverse effects such as Cushing’s syndrome, growth suppression, osteoporosis, and adrenal insufficiency, while many patients with severe asthma show poor treatment response due to steroid resistance [20,21,22,23]. Nanotechnology presents a transformative solution by enabling targeted, sustained, and precise drug delivery directly to the inflamed airways. Utilizing nanocarriers such as liposomes, polymeric nanoparticles (NPs), and metallic NPs, this approach enhances corticosteroid efficacy, mitigates oxidative stress, and minimizes systemic side effects. Furthermore, gene-loaded and siRNA-loaded nanocarriers, along with nano-biosensors, provide opportunities for immune modulation, early disease detection, and improved management of corticosteroid-resistant asthma [24,25,26,27,28,29,30,31]. This review aims to explore the potential of nanozymes as innovative therapeutics for asthma while also addressing the limitations of current corticosteroid-based treatments. It focuses on how nanozymes regulate redox imbalance and modulate immune responses to alleviate airway inflammation and oxidative stress. This review also highlights nanozymes as a promising redox-responsive and immunomodulatory strategy for improving asthma management and treatment outcomes.

## 2. Pathophysiology of Asthma

Asthma is a heterogeneous, chronic inflammatory disease [32]. It has a direct impact on approximately 260 million individuals worldwide, according to the Global Initiative for Asthma (GINA 2024). Wheezing, elevated chest pressure, coughing, dyspnea, and restricted expiratory flow (WHO) were all signs of asthma. Th2-high versus Th2-low asthma can be distinguished by their prevalence. Th2-high is the most abundant and is usually associated with hormones, allergens, exercise, and systemic inflammation [33,34]. Th2-low is usually found during infections, including viral and certain bacterial strains [34]. Increases in innate and adaptive immune cell populations, such as Th1 cells, neutrophils, and macrophages, are a feature of Th2-low inflammation. Among the main inflammatory mediators generated in Th2-low inflammation are IFN-γ, IL-1β, and TNFα [35]. The first epithelial cells that are affected in the airway produce cytokines such as (L-12, IL-8, IL-6) and chemokines C-C Motif Chemokine Ligand 5 (CCL5) and C-X-C Motif Chemokine Ligand (CXCL9, CXCL1) [36]. These mediators activate dendritic cells (DCs), which, in the presence of IL-12 and IFN-α, drive the differentiation of naive CD4 into Th1. After differentiation, Th1 cells secrete IFN-γ, which activates the Janus Kinase–Signal Transducer and Activator of Transcription (JAK-STAT1) signaling pathway through the interferon gamma receptor (IFNGR) on various cells within the airway [34]. That signal enhances the expression of a gene that is associated with inflammation and raises the expression of an adhesion molecule that adds more immune cells in the inflamed area [37]. IFN-γ also inhibits the differentiation of Th2 cells [34]. IFN-γ contributes to stimulating airway smooth muscle (ASM), which releases CXCL1, CXCL2, CXCL3, and CXCL8 that enhance neutrophil cells [32,34,36,37,38]. In the presence of IFN-γ and lipopolysaccharide (LPS), the macrophages differentiate into classically activated macrophages (M1) that release IL-12, TNF-α, IL-6, and reactive oxygen species (ROS), leading to tissue damage [39]. Another cytokine involved in Th2-low asthma is TNF-α, which is produced by M1 macrophages and Th 1. TNF-α binds to its tumor necrosis factor receptor (TNFR1 and TNFR2), initiating the downstream pathways such as NF-κB and Mitogen-Activated Protein Kinase (MAPK) that lead to increased levels of pro-inflammatory cytokines, especially IL-6 and chemokines (e.g., IL-8), which attract neutrophils.

**Figure 1:**
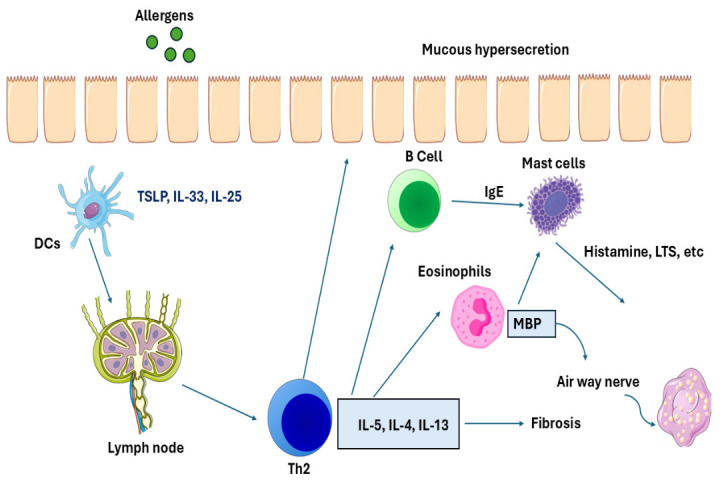
Cellular pathogenesis of asthma: high T2-inflammation. Allergen exposure at the epithelial cell of the airway stimulates the dendritic cell to release thymic stromal lymphopoietin (TSLP), IL-25 and IL-33 and migrate to lymph nodes to activate the differentiation of naive T cells (T0) to T2. These release IL-4, IL-5 and IL-13 that stimulate B cells to export IgE and they stimulate eosinophils to release MBP. IL-4, IL-5 and IL-13 stimulate fibrosis. Ultimately, these lead to mucosal hypersensitivity, airway remodeling and the rest of the features of asthma.

Furthermore, asthmatic T2-high inflammation is the most common [40]. In this type, the patient is termed atopic (genetic predisposition) [35]. Initially, the epithelial cells are exposed to allergens, which then release (IL-25, IL-33, and TSLP) and stimulate the differentiation of DCs [41,42]. These stimuli cause the release of a range of chemoattractants, such as CCL20, the ligands for CCR6 that also stimulate DC maturation, and then DCs pick up the allergens and process them [42]. then, mast cells express both major histocompatibility complex (MHC) class I and class II molecules on their surface, enabling them to present antigens and interact with CD8^+^ and CD4^+^ T cells, respectively, in the lymph node. Then naive T cells differentiate into T2 cells that release IL-5, IL-4, IL-13 and IL-9. IL-13 and IL-4 stimulate the B cells to release IgE which increases mucous production and airway remodeling [8], as illustrated in Figure 1. Also, IL-5 stimulates the eosinophils to release major basic protein (MBP), eosinophil cationic protein (ECP), and cysteinyl leukotrienes (CYS-LTS), which leads to tissue damage and bronchial hyperresponsiveness [43]. IL-9 stimulates mast cells, and then the IgE binds to high-affinity IgE receptors (FcεRI), which are found on the surface of mast cells and other cells such as basophils, leading to an increased response by the mast cells [41,44]. At this point in the process, no symptoms are present. Some mediators are released immediately, such as histamine; it causes bronchoconstriction, leading to shortness of breath, vasodilation of blood vessels and increased mucus production. Mast cells also release proteases such as tryptase and chymase, which damage tissue, cause extracellular matrix remodeling and amplify the inflammatory response. In addition, platelet activation factor (PAF) is released which attracts more inflammatory cells. Another group of mediators is released approximately one minute to one hour after activation of mast cells: CysLTs, including LTC4 and LTD4. These have a 100-fold greater potency than histamine in stimulating the formation of mucus, contraction of smooth muscle in the airways and drawing in additional inflammatory cells such as eosinophils. Prostaglandin D2 (PGD_2_) attracts inflammatory cells and causes bronchoconstriction. A vicious cycle of inflammation is created by cytokines such as TNF-α, IL-4, IL-5 and IL-13, which recruit and promote the Th2 response [41,42,45].

## 3. Fundamentals of Nanozymes and Functional Diversity

### 3.1. Nanozymes

Natural enzymes are used in various industries, including the food, medical and biofuel industries. However, they can be affected by temperature, pH and ionic strength, leading to protein denaturation and functional loss. Their practical applications are limited by high preparation, purification and storage costs, prompting an interest in developing stable, affordable substitutes with similar catalytic properties [46]. Nanozymes are NPs that exhibit catalytic activities mimicking the biological activity of enzymes. They have the unique physicochemical characteristics of nanomaterials as well as the specific catalytic activity of enzymes [47]. The name “nanozyme” was used in 2004 to describe transphosphorylation catalysts based on gold NPs [48,49]. A 2007 study by Gao and colleagues found that the spontaneous peroxidase-like enzyme activity of magnetite Fe_3_O_4_ NPs was 40 times higher than that of horseradish peroxidase (HRP) at the same molar concentration [50]. According to recent studies, although Fe_3_O_4_ nanozymes may exhibit higher apparent turnover numbers than HRP in model peroxidase assays, they lack well-defined catalytic active sites. As a result, nanozymes generally indicate lower substrate affinity and diminished overall catalytic efficiency compared with natural enzymes [51]. The greater catalytic activity of iron oxide NPs (Fe_3_O_4_) as opposed to HRP was thought to be caused by the quantity of surface ferrous and ferrite ions [50]. After this study, research in this field exploded, and many metal and metal oxide-based nanozymes with catalase, oxidase, peroxidase and superoxide dismutase-like activity were reported [52,53,54,55,56,57]. The production of reactive oxygen radicals or electron transfer mechanisms has been cited as the primary cause of nanozyme catalytic activity.

### 3.2. Nanozyme Composition

**Figure 2:**
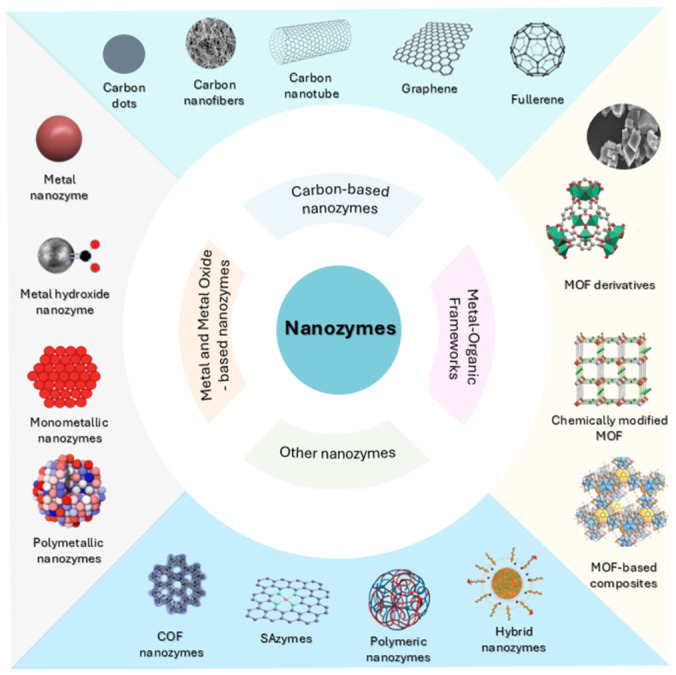
Various types of nanozymes.

Nanozymes’ catalytic activity is mostly determined by the fundamental characteristics of the materials used. Thousands of different kinds of nanomaterials have been created and investigated, whether metallic or non-metallic, organic or inorganic. The size of the nanozymes spans from the nanoscale down to the single-atom and is nevertheless capable of exhibiting catalytic qualities similar to those of large enzymes [47]. The most common types of nanozymes in terms of material matrix are single-atom (SAzyme), metallic, carbon-based nanozymes and metal–organic frameworks (MOF) [47], as illustrated in Figure 2.

#### 3.2.1. Metal-Based Nanozymes

To date, nanozymes have been at least mentioned in more than 2877 research publications. Nanozymes based on metals are the most prevalent type of nanozyme matrix. There are two major types of metal-based nanozymes: iron-based and non-iron-based [47]. Fe-based nanozymes [50] mainly include iron oxide NPs, iron phosphates, iron chalcogenides, Prussian blue and other related cyanometallate frameworks [58,59]. Non-iron nanozymes are mainly composed of noble metal nanomaterials such as Ag, Au, Pt and Ir as well as numerous multimetallic structures, including oxides and sulfides of Ce, V, Mn, Cd and Cu [60,61,62,63]. Due to their adaptable and controllable structure, shape and oxidation state, metal-based and functionally modified hybrid nanomaterials have recently emerged as the most studied class of nanozymes. The SnFe_2_O_4_ nanozyme that exhibited catalase-like and glutathione peroxidase-like activity was prepared by Feng et al. [64] and modified with a copolymer with polystyrene and polyethylene glycol (PS-PEG) to enhance its water dispersibility and overall biocompatibility. Li and colleagues synthesized an Fe-core/Fe_3_O_4_-shell covered with Cu_2−x_S nanozyme, adding β-lapachone to increase ROS generation and stop tumor growth [65]. The metal morphologies and compositions significantly affect the variety and activity of nanozymes. Because of the synergistic and electrical effects, multi-metallic nanozymes usually show better catalytic activity than single metal components. Enhancing catalytic efficiency through sensible management of the quantity of metal components and architectures is one of the key objectives in the study of metal-based nanozymes [66,67]. In order to enable Au–Pt bimetallic nanostructures to function as nanozymes capable of driving the glucose cascade reaction, Cai and colleagues [68] designed them with exquisite structural control, resulting in segregated, alloyed, and core–shell topologies.

#### 3.2.2. Carbon-Based Nanozymes

Carbon-based nanomaterials such as carbon nanotubes, fullerenes, graphene, carbon dots (C-dots), and graphdiyne, together with their various doped forms, are examples of carbon-based nanozymes that were initially described in 2010 [69]. Due to their high stability, low cost, and ease of fabrication, these nanozymes have found extensive use in sensing, treatment, and catalysis (CAT). Graphene oxide, which exhibits peroxidase (POD)-like activity that is employed for glucose monitoring, was discovered by Song et al. [70]. Graphdiyne oxide’s (GDYO) effective POD-like activity was demonstrated by Mao et al. [71]. This activity is mainly due to superoxide anion (O_2•_^−^) production from hydrogen peroxide (H_2_O_2_) breakdown. Functional groups that contain oxygen have crucial roles in such nanozymes.

Fan et al. used a solvothermal technique to produce C-dots with strong superoxide dismutase-like (SOD) activity [72]. They passivated functional groups in order to study the mechanism of action of C-dots. H bonds with carboxyl, hydroxyl, and amine groups held superoxide to the surface region of the C-dots. The intermediate product was stabilized by the reduced C-dots and oxygen generated by the electron-deficient structure. Carboxyl, hydroxyl, and amino groups were associated with activity similar to SOD.

#### 3.2.3. MoF-Based Nanozymes

In 2017, it was initially found that MOFs, a new and distinct type of hybrid porous crystalline materials combining organic and inorganic elements, had nanozyme activity [73]. Their many channels and porous structure allow small molecular substrates to reach and have complete access to the catalytic sites, which is advantageous for product diffusion and transportation. The catalytic reaction which exhibits size selectivity, allows fine control over the molecular size of the substrates and depends on the highly uniform sizes and pore shapes of MOFs. Furthermore, a variety of metal-based nodes and organic ligands, along with multifunctional alterations, can improve their usefulness, biocompatibility, and biodegradability [74,75]. Because of these features, MOFs can display a variety of enzyme-like qualities in biosensing, biocatalysis, and biomedical domains. MOF-based nanozymes have been extensively characterized for their catalytic activities and physicochemical properties. While preclinical or clinical studies in asthma are scarce, these materials hold potential for application in airway inflammatory diseases, providing a basis for future research.

#### 3.2.4. SAzymes

SAzymes are revolutionary high-performance nanozymes that have garnered a great deal of research interest since they were initially reported in 2019 [76]. A small number of active sites on the nanomaterial’s surface, including edge atoms and defect sites where coordination is unsaturated, are primarily responsible for the activity of conventional nanozymes. Their activity is significantly lower than that of natural enzymes due to the difficulty in identifying and quantitatively controlling these sites, which also makes the catalytic process exceedingly complex. Scaled to the atomic level, NPs can display electrical and geometric properties that differ greatly from those of NPs. They exhibit a straightforward atomic structure, distinct coordination frameworks, reactive sites, catalytic processes, and exceptional catalytic activity, in addition to possessing the greatest atomic utilization rates. With the metal atoms individually distributed throughout the supports, SAzymes, a kind of single-atom nanomaterial capable of mimicking catalytic activity, have effectively overcome the difficulties and demonstrated very high enzyme-like activity [47]. When compared to NPs, the special properties of SAzymes result in the highest rates of atom usage and give them distinct geometric and electrical architectures. The primary structure of SAzymes is M-N-nC, and the specific core atoms and their coordination environment dictate the SAzymes properties. Considering M-N-nC, M stands for the core of metal; the specific metal and oxidation state of the metal have an impact on the metal center, which is the center of catalytic activity. N stands for the coordination state of nitrogen; coordination connections between nitrogen atoms and the metal core stabilize the SAzymes and change their electronic density. The carbon matrix is denoted by the letter C. As carriers, carbon-based materials give the metal core and coordinating atoms a stable platform. For the desired SAzyme activity and selectivity, the appropriate surrounding coordination of the central metal in the M-N-nC framework is essential [77,78,79,80,81,82,83]. SAzymes’ physicochemical characteristics and catalytic activity have been extensively researched. They show promise for application in airway inflammatory disorders and may encourage future research efforts, despite the lack of preclinical and clinical data in asthma.

### 3.3. Properties of Nanozymes

To date, numerous nanomaterials with exceptional enzyme-like properties have been found. Oxidoreductases and hydrolases are the primary types of enzymatic activity [84], but the majority of the catalytic mechanisms have not been fully investigated [85].

#### 3.3.1. Peroxidase Activity

The first catalytic action to be clearly confirmed was the POD-like activity, which has been reported across several types of nanomaterials such as metals, metal oxides, and carbon-based nanozymes [85]. These nanozymes can promote substrate oxidation, and their efficacy varies based on parameters like pH, temperature, and substrate concentration in a manner similar to natural peroxidase. Peroxide (usually H_2_O_2_) and an oxidized substrate are the two substrates that are typically present in the process [86].

Halide, thiol, and glutathione are special substrates that are necessary for some nanozymes to finish their catalytic reaction [87]. Numerous species include POD, which frequently oxidize substrates such as proteins, lipids, polysaccharides, and nucleic acids. POD mimics’ catalytic pathway mostly entails electron transport and ROS production. Ferrous ions on the surface of Fe_3_O_4_ NPs are primarily responsible for activating H_2_O_2_ and producing ROS, making them the earliest proposed nanozymes which naturally exhibited POD-like activity [85].

#### 3.3.2. Oxidative Activity

Molecular oxygen and, in some cases, other oxidizing agents can help oxidases in driving substrate oxidation, resulting in the formation of oxidized products in addition to H_2_O/H_2_O_2_/O_2_ [88]. Likewise, nanomaterials such as Ru, Au@Pt, cerium dioxide (CeO_2_), and N-CNMs have been found to have the capacity to oxidize different substrates, and their enzymatic activity is highly dependent on substrates, pH, and temperature [85]. Being crucial stages in the reaction cascade, POD-like activity in nanozymes often entails producing reactive species and promoting electron transfer. Nanozymes serve as both the recipient and the transmitter of electrons during the electron transfer process. The hydrated glucose anion first attaches to the gold surface, forming electron-rich gold sites. These sites subsequently use nucleophilic attack to activate dissolved oxygen, creating a dioxo-gold intermediate that moves electrons from glucose to molecular oxygen. This method reveals how bare gold NPs (AuNPs) display POD-like catalytic behavior [85].

#### 3.3.3. Catalase Activity

In nature, H_2_O_2_ is a necessary ROS. CAT-like nanozymes can catalyze H_2_O_2_ to produce molecules of oxygen and water. This reaction is dependent upon the concentration of both nanozymes and substrates, and the higher the concentration of either, the higher the decomposition rates [89]. By preventing the buildup of ROS, this method can effectively protect cells from oxidative damage. The CAT mimicking activity is usually measured by the amount of oxygen that is dissolved, or oxygen available throughout the reaction process [90,91]. The pH and temperature affect the activity of nanozymes, which mostly favor neutral and alkaline conditions. In somewhat acidic conditions, some show CAT-like activity [90,91]. Their catalytic mechanism is Michaelis–Menten in character [92]. The shape, structure, and valence state at the surface of NPs, as well as the pH of the environment around them, are key factors that strongly influence the catalytic reactions associated with CAT-like activity. At the time of writing, very little is known about the catalytic mechanisms of nanozymes. They often involve redox reactions and adsorption activation. Metal-based nanozymes that mimic CAT activity follow a distinct catalytic pathway which is tightly linked to chains of free radicals [85].

#### 3.3.4. Superoxide Dismutase Activity

Destructive ROS are extensively present in cells and include superoxide anions such as HOO_•_ and O_2•_^−^ (O_2•_^−^ is the primary type) [85]. They have the ability to start a cascade of reactions that release more damaging free radicals. Although superoxide radicals generally decompose on their own under normal conditions, the decomposition rate is incredibly slow [85]. In nature, SOD is a viral antioxidant that breaks down superoxide anions into H_2_O_2_ and O_2•_ Due to their ability to eliminate O_2•_^−^, certain nanozymes (Pd, MnO_2_, PB, and fullerene) are thought to be promising alternatives for SOD [82]. When nanozymes have more than one enzyme activity, SOD activity takes over due to the pH of the surrounding environment or the nanomaterial’s surface ions and structure. A greater electrostatic barrier to superoxide radicals caused by an improper pH inhibits catalytic activity [93]. These reactions occur via a ping-pong process, in a manner similar to natural enzymes. Electron transfer and substrate adsorption are the main contributions of nanozymes enabled by surface unpaired electrons and multivalent ions [85]. However, pH, surface ion content, or nanozyme structure determine the actual mechanism followed. Superoxide proton formation and surface-mediated HOO_•_ rearrangement are essential processes for metal-based SOD-mimicking nanozymes.

#### 3.3.5. Hydrolase

One typical enzyme activity, hydrolase, mediates the breaking of chemical bonds through hydrolysis, which is essential to biological systems. Recent research has found that a variety of nanomaterials including Au, CeO_2_, Ce-AuNPs, MOFs, Fe_3_O_4_, and ZrO_2_ have exceptional hydrolase-like activity [85]. Bond breaking and free radical formation are strongly related, despite the fact that little research has been done on the catalytic mechanism of hydrolase-like activity in nanozymes [85].

Within double-stranded DNA molecules containing over 90 base pairs, Sun and colleagues found that chiral cysteine-modified NPs could precisely detect and cut at the restriction site GAT′ATC by mimicking a restriction endonuclease [94]. In this process, NPs acted as donors of electrons to produce ROS, while DNA acted as an acceptor of electrons to break the phosphodiester link [94]. Asymmetric copper sulfide quantum dots (d/L-Ds) were used as photocatalysts to disrupt proteins in their previous investigation [95]. Proteins were cleaved at specified places (61 KD + 5 KD) as a result of the peptide link between amino acids K and L being broken by the OH generated by d/L-QDs [95].

#### 3.3.6. Diversity of Nanozyme Activity

Nanozymes’ numerous enzyme-like functions are their most important characteristic. Even if the catalytic activity of various nanomaterials is identical, their catalytic pathways are entirely dependent on the pH of the surrounding environment, as well as the size, surface ion states, and shape of the nanomaterials [85].

There are about 20 distinct types of nanozymes that functionally resemble many enzymes, such as those possessing bi-, tri-, tetra-, and penta-enzyme-like functions. Under acidic circumstances (pH 4.8), iron oxide NPs were discovered to demonstrate POD-like activity by catalyzing H_2_O_2_ to generate hydroxyl radicals (_•_OH) [55]. Additionally, iron oxide NPs demonstrated CAT-like activity by catalyzing H_2_O_2_ to O_2_ in both neutral and alkaline environments [55]. The class of multi-enzyme-like nanozymes that has been investigated the most is the oxidase (OXD)-POD-like group. The defining characteristic of the OXD-POD nanozyme is its capacity to create a cascade catalytic system by combining the capabilities of OXD to convert O_2_ to H_2_O_2_, while POD consumes H_2_O_2_ to create _•_OH. Bi-enzyme-like action of OXD-POD was mostly observed in GOX-POD, cholesterol oxidase–peroxidase (COX-POD). Additionally, Dong et al. discovered that Co_3_O_4_ nanoplates have POD-CAT-like activity at the same time [94]. The pH could be used to switch between these two types of enzymic activities. Additionally, the pH shift and the dual-enzymatic activity were significantly impacted by temperature and Co_3_O_4_ concentrations [94]. Two cascade catalytic processes could be created by nanozymes with tri-enzyme-like activity. For instance, nanozymes that resemble OXD-CAT-POD can create two different types of cascade catalytic systems: OXD-POD and CAT-OXD [53]. The nanozymes catalyzed the production of ^•^HO and O_2_ by using their POD-CAT-like activity. Its OXD-like action further uses O_2_ to produce O_2•_^−^.

## 4. Nanozyme Platforms Relevant to Asthma Model

Chronic inflammation of the airways, tissue damage, structural remodeling, and airway hyperresponsiveness (AHR) are the key features of asthma [96]. The oxidative reactions that cause inflammation are triggered by environmental stimuli such as tobacco smoke, allergens, and air pollution. Oxidative stress is exacerbated when this inflammation attracts eosinophils, neutrophils, and macrophage cells that are also significant ROS generators [97].

Building upon the classification and catalytic properties of nanozymes discussed previously, their therapeutic relevance in asthma largely depends on how specific enzyme-mimicking activities interact with key pathological features of the disease, including oxidative stress, immune dysregulation, and airway remodeling. Nanozymes can effectively scavenge ROS, reduce oxidative stress, and subsequently attenuate inflammation by imitating natural antioxidant enzymes [98].

In a recent study, the synthesis of multifunctional nanogels was carried out; catalase-loaded nanogels (M-CAT-NGs) were developed with excellent antimicrobial and anti-inflammatory properties. In vitro experiments confirmed their ability to reduce ROS production and downregulate IL-1β, IL-6, and TNF-α expression. In vivo experiments showed M-CAT-NGs effectively accumulate in the lungs, improving AHR, inhibiting neutrophil accumulation, reducing ROS levels, and attenuating airway inflammation. These results demonstrate their encouraging potential for the treatment of neutrophilic asthma [99].

In addition to redox regulation, nanozymes also contribute to targeted immunomodulation, further enhancing their therapeutic efficacy. Another study developed a diselenide-bridged mesoporous organosilica NP stabilizer (SeMSNs@CS@Ap), which represents a significant advancement in precision medicine for asthma treatment [100]. This nanozyme inhibits allergen-triggered immune responses in asthma by targeting mast cell surface receptors with surface-conjugated IgE aptamers, achieving up to 78% efficacy. Its diselenide backbone responds to ROS in inflammatory conditions, enabling the release of a stabilizing agent and providing anti-inflammatory and immunosuppressive effects. Demonstrating sustained therapeutic benefits for up to 28 days without side effects, this system enhances vascular permeability in asthmatic mice, representing a novel strategy for allergic asthma management [100]. Moreover, another study demonstrated that surfactant-dependent copper sulfide NPs (CuS NPs) represent a profound fusion of therapeutic and diagnostic roles. These nanozymes significantly improve accurate monitoring of airway inflammatory status by increasing adrenaline detection sensitivity to 0.12 µM by the synergistic utilization of POD and SOD mimetic activities. At the same time, CuS NPs prevent bronchodilators from being broken down by POD, increasing their bioavailability by around 2.3 times. CuS NPs achieve an 83.6% **·**OH scavenging efficiency in asthma treatment models, directly attenuating important clinical indications of airway remodeling, highlighting their dual benefit in oxidative stress prevention and precise drug delivery [101].

Overall, there is currently insufficient research into this topic, which emphasizes the need for researchers to focus more on this area. This subject is quite important, particularly when it comes to employing nanotechnology to cure asthma with the use of nanozymes. The main areas of progress in the treatment of asthma have been precision medicine, biologics, endotypes, phenotypes, and biomarkers. Personalized treatment requires an understanding of asthma biomarkers, phenotypes, endotypes, genetics, and regional patterns. Through the regulation of drug release, nanotechnology can improve focused therapy. For patients with asthma of the Th2-high and Th2-low subtypes, future research may employ medications based on NPs that target the release of neutrophils or eosinophils, improving the efficacy of treatment. The treatment of asthma is anticipated to evolve toward this individualized approach.

### 4.1. Mechanistic Relationship Between Acute Lung Injury and Asthma

Acute lung injury (ALI) and asthma are both inflammatory lung diseases but of distinct clinical and immunological character. ALI and its severe form, acute respiratory distress syndrome (ARDS), are life-threatening conditions marked by neutrophilic alveolitis, disruption of the alveolar–capillary barrier, uncontrolled inflammation, and progressive hypoxemia, with mortality rates exceeding 40% [102,103]. In contrast, asthma is an airway disease marked by chronic inflammation and hyperresponsiveness. Increased airway bronchoconstriction, immune cell accumulation including lymphocytes and eosinophils, mucus hypersecretion, pro-inflammatory mediators such as cytokines, histamine, and prostanoids, and reactive oxygen species are all part of its pathophysiology [104].

Despite these variations, the two conditions have two important characteristics in common: an oxidative stress–inflammation cycle that contributes to tissue damage and an overabundance of ROS. For example, neutrophils and macrophages in ALI and eosinophils/neutrophils in asthma produce high levels of O_2•_^−^, H_2_O_2_, and other ROS through NADPH oxidase and peroxidases [104,105]. These ROS trigger common signaling pathways that cause pro-inflammatory cytokines and barrier dysfunction, such as NF-κB and MAPKs, as demonstrated in Table 1 [102]. Significantly, similar molecular processes cause ROS-driven damage to the alveolar–capillary barrier in ALI and to the airway epithelium in asthma, establishing a mechanistic connection between the two conditions [104,105].

Overall, the shared ROS–inflammation axis between asthma and ALI suggests that antioxidant strategies, such as catalytic nanozymes, may be useful in both contexts, providing a mechanistic basis for cautiously extrapolating nanozyme effects from one disease to the other. As shown in Table 1, several nanozyme platforms investigated in ALI models demonstrate potential relevance to asthma therapy.

## 5. Role of Oxidative Stress in Asthma and the Potential of Nanozymes

Reactive oxygen species are chemicals that contain unstable oxygen atoms due to the presence of unpaired electrons (free radicals) from oxygen, so they are very reactive in affecting proteins, lipids, and DNA inside the cell. Reactive oxygen species cause oxidative stress which is associated with asthma and pulmonary hypertension. pH [110,111]. Examples of ROS that exist in the body include the following:

1. O_2•_^−^;
2. Hydrogen peroxide (H_2_O_2_);
3. Hydroxyl radicals (_•_OH).

**Figure 3:**
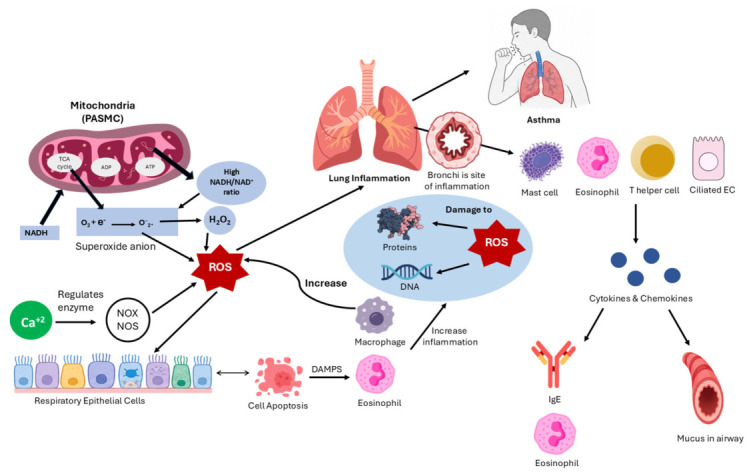
Production of reactive oxidative stress and inflamed airways. This diagram illustrates the mechanisms by which ROS and related chemicals cause pulmonary inflammation, ultimately leading to asthma. ROS are produced by mitochondria in PASMCs through several processes, including oxygen reduction and the action of NAD/NADH. Calcium also regulates enzymes such as NOS/NOX, which increase ROS. These processes lead to the accumulation of ROS in cells, causing apoptosis of the cells. These cells send signals to the immune system, causing an increase in ROS through the interaction of immune cells with each other, such as eosinophils and mast cells. Ultimately leading to the secretion of mucus and predisposing to pneumonia.

Mitochondria are a major source of ROS production, including pulmonary artery smooth muscle cells (PASMCs) [112,113]. The main reaction that produces ROS is the reduction of oxygen by one electron to superoxide, and this occurs in an oxygen-rich environment. Mitochondria also produce H_2_O_2_, and if the NADH/NAD+ ratio in the mitochondria is high, they produce ROS [113,114]. Also, calcium/Ca^2+^ works to produce ROS by regulating some enzymes that generate ROS, such as NADPH oxidase (NOX) and nitric oxide synthase (NOS) [115,116]. ROS are present in the body during cellular respiration in mitochondria in low normal amounts, but they increase through the immune system’s response to bacteria and viruses. In asthma, they contribute to the chronic inflammation in the bronchi, which categorizes asthma into eosinophilic and neutrophilic asthma, as illustrated in Figure 3. Certain conditions can precipitate asthmatic exacerbations, including exercise-induced asthma, occupational asthma caused by industrial irritants such as fumes, gases, or dust, and allergy-induced asthma [117,118]. Asthma results from the interaction of several factors including mast cells, eosinophils, epithelial cells, T helper lymphocytes, and respiratory tract muscles. This interaction causes the secretion of cytokines and chemokines, which are inflammatory molecules. Cytokines stimulate the production of allergy-associated IgE antibodies and attract acidic cells such as eosinophils, leading to mucus secretion and reduced antioxidant activity [119].

### 5.1. ROS in Airway Epithelial Injury and Immune Activation

Epithelial cells are linked together by cohesive bonds called junctional complexes which, when damaged, increase permeability. This increased permeability allows foreign organisms to enter, for example, the lungs and cause infection and inflammation and then activate the immune system. Allergens such as dust, cigarette smoke, some foods, and air pollutants increase ROS, leading to oxidative stress. Naturally, the body is in a state of dynamic equilibrium between oxidants, such as ROS, and antioxidants. An increase in oxidants and a decrease in antioxidants within the respiratory tract causes oxidative stress that leads to the death of epithelial cells and damage to DNA and mitochondria [120,121]. Increased apoptosis, especially in ciliated epithelial cells, leads to the loss of barrier function and increased permeability; this relates to the role of ROS in activating immunity. Cells affected by reactive oxygen species and allergens secrete cytokines and chemokines such as IL-25, IL-33, and TSLP, which activate immune cells such as mast cells and Type II innate lymphoid cells “ILC2” which act against fungal cells. Mast cells secrete histamine, which causes bronchial constriction, and leukotrienes, which increase mucus production and smooth muscle contraction. Subsequently, chemokines attract eosinophils and type II Th2 that secrete IL-4, IL-5, and IL-13, which promote IgE production and increase inflammation. Neutrophils and macrophages that arrive in the lung produce ROS and degrading enzymes such as proteases. When mitochondria are damaged by ROS, they secrete danger signals known as damage-associated molecular patterns (DAMPs); these signals move from inside the cell to the extracellular space, alerting the immune system to the presence of danger [121,122,123]. M1 macrophages are usually inflammatory, while alternatively activated macrophages (M2) are anti-inflammatory and associated with wound healing and tissue reconstruction [123]. M2 macrophages are predominant in the lung if there is an allergic type of asthma caused by dust mites, while in NAA, M1 macrophages are present in the lung [124]. M1 macrophages play a role in strongly enhancing asthmatic pathophysiology and, accordingly, making the response to treatments weak [125].

### 5.2. Nanozymes Restoring Redox Balance, Inhibiting Neutrophil Infiltration

In recent years, the term “nanomedicine” has emerged. Its central theme is converting active ingredients in traditional medicines into NPs, potentially making them safer. They precisely target diseased tissues, reduce damage to healthy tissues, and ensure the medicine reaches its target cells [126]. According to the nanozyme classification, AuNPs can be formally classified as metal-based peroxidase-mimicking nanozymes due to their intrinsic enzyme-like catalytic activity. AuNPs have anti-inflammatory and antioxidant properties and have been associated with treatment for cancer, sepsis, and rheumatoid arthritis [127,128,129,130]. A study was conducted that showed injecting AuNPs with 13 nm citrates through the nose works to alleviate asthmatic symptoms in mice sensitive to corticosteroids through a mechanism that works to reduce the level of ROS and reduce the production of cytokines and chemokines [131]. AuNPs in asthma work to inhibit some of the pathological changes associated with this condition, such as airway hyperreactivity, inflammation, and mucus overproduction. This occurs by maintaining key pathways involving histone deacetylase 2 (HDAC2) and nuclear factor erythroid 2-related factor 2 (NRF2). These vital pathways work on anti-inflammatory responses, prevent ROS, and restore oxidative balance [132]. Medicinal mushrooms are believed to have therapeutic importance and benefits such as liver protection, antioxidants, and antitumor properties. An active ingredient extracted from the chaga mushroom, called beta-glucan (BG), is a high-molecular-weight sugar. The result of using this mushroom on asthma patients was that it reduced the entry of inflammatory cells into the respiratory tract, lowered the level of IgE in the blood, reduced DNA damage, lowered the level of oxidative stress (malondialdehyde, MDA, and ROS), increased antioxidants such as glutathione peroxidase (GPX) and glutathione (GSH), and also increased the activity of the glutathione peroxidase 4 (GPX4) gene responsible for reducing iron deposited in the lungs [133].

### 5.3. Nanozymes in Ferroptosis Modulation for Asthma

Ferroptosis is a type of regulated cell death associated with lipid peroxidation and intracellular iron accumulation, leading to oxidative stress. Ferroptosis is precisely regulated by several signaling pathways, including those related to iron, lipid, and amino acid metabolism [134]. Currently, a growing body of research indicates that ferroptosis plays a role in asthma. Its activity is controlled by metabolic and signaling elements, including lipid metabolism enzymes, GPX4, and the cystine/glutamate antiporter (system Xc-). Tissue damage and airway inflammation may worsen if these pathways are dysregulated [135].

Important biochemical changes are linked to ferroptotic events in asthmatic airways. Signals like lipid peroxides (like MDA and 4-HNE) and DAMPs (like HMGB1 and IL-33) activate immune cells, such as Th17 cells and M1 macrophages. By releasing cytokines (like IL-6 and TNF-α) and ROS, activated immune cells further disturb iron homeostasis (like IRP2 activation and ferritinophagy) and encourage lipid remodeling (like ACSL4 upregulation), which intensifies ferroptosis and maintains the inflammatory cycles that are indicative of severe asthma phenotypes [135].

Ferroptosis-associated pathways have been identified in immune cell populations and airway epithelial cells. Ferroptosis inhibitors, such as liproxstatin-1 and ferrostatin-1, which target iron metabolism and lipid peroxidation, have shown notable effectiveness in a number of preclinical asthma models. Because of their strong capacity to scavenge lipid radicals, these substances reduce important pathological characteristics such as inflammatory cell infiltration, airway hyperreactivity, and structural remodeling. They minimize the loss of epithelial barrier function and consequent amplification of inflammation by stopping membrane peroxidation chains [135].

By mimicking the actions of natural enzymes like CAT and SOD, nanozymes can transform harmful O_2•_^−^ into innocuous oxygen, lowering inflammation and avoiding ferroptosis. Additionally, it can further prevent ferroptosis by upregulating the expression of GPX4, a crucial enzyme in the antioxidant defense system [136]. Nanozymes provide a dual output mechanism of ferroptotic damage mitigation and stronger antioxidant defenses by preventing excessive buildup of ROS, which can trigger lipid peroxidation, and promoting wide-ranging cellular antioxidant capacity. Such a dual redox modulation is specifically applicable to asthma, in which inflammatory signaling and tissue damage are enhanced by iron-mediated lipid peroxidation.

### 5.4. Nanozymes in Targeting Inflammasome Levels in Asthma

The NOD-like receptor family pyrin domain-containing 3 (NLRP3) inflammasome is a cytosolic multiprotein complex that identifies pathogen-associated molecular patterns (PAMPs) and DAMPs. When NLRP3 is activated, caspase-1 is activated, leading to the release of IL-1β, IL-18, and gasdermin D, which induce pyroptotic cell death [137]. This route, which is important in innate immunity, may be involved in pediatric asthma pathogenesis, especially in neutrophilic corticosteroid-resistant patients. Airway inflammation, neutrophil recruitment, and airway hyperresponsiveness are all correlated with elevated NLRP3 expression and activity, highlighting the protein’s potential as a therapeutic target outside of the traditional corticosteroid therapy strategy [137]. According to research, excessive ROS production in the airways of asthmatics causes oxidative stress and chronic inflammation [138]. ROS plays a critical function in activating the NLRP3 inflammasome in bronchial epithelial cells. Thioredoxin-interacting protein (TXNIP), an NLRP3 ligand, reacts to ROS. Normally, thioredoxin (TRX) binds to TXNIP and inhibits its activity. Elevated ROS levels promote TRX-TXNIP dissociation, which allows TXNIP to link with the leucine-rich repeat domain of NLRP3, initiating inflammasome activation [138]. Experimental models of allergic airway disease have established that mitochondrial ROS is a critical activator of the NLRP3 inflammasome and that its decrease reduces inflammation and hyperresponsiveness [139].

Nanozymes, which are nanomaterials with intrinsic enzyme-like antioxidant capabilities, provide a promising technique for modulating inflammasome activation by scavenging ROS and lowering the oxidative triggers that induce NLRP3 assembly [140]. For example, cerium oxide nanozymes can modify cellular microenvironments. These NPs replicate the actions of natural enzymes like CAT, SOD, and peroxidase, allowing for the neutralization of ROS via numerous pathways. CeO_2_NZs are unusual in their ability to transition between Ce^3+^ and Ce^4+^ oxidation states, allowing for continual regeneration and persistent ROS scavenging, demonstrating their capacity to indirectly reduce inflammasome activation and downstream cytokine release in inflammatory settings [140]. For instance, in other disease models, CeO_2_-based nanozymes have been demonstrated to reduce oxidative stress and restore GPX4 expression by scavenging ROS and lowering lipid peroxides, which in turn suppresses ferroptosis and related inflammation [141]. Ce-based nanozymes with phosphatase-like activity offer a stable and precisely controlled approach for mast-cell stabilization in asthma treatment. Through Ce^3+^/Ce^4+^ redox cycling and oxygen-vacancy-facilitated P–O bond hydrolysis, they continuously inhibit phosphorylation signaling pathways and the release of allergic mediators, demonstrating superior efficacy compared with traditional stabilizing agents [142]. Also, there is another notable example of a newly developed class of polydopamine (PDA)-coated CeO_2_ nanozymes (Ce@P), which act as nanozymes by mimicking antioxidant enzyme functions and are powerful ROS scavengers, scavenging intracellular ROS and reducing inflammatory responses to ALI. It was discovered that combining Ce@P with NIR irradiation could enhance its ROS scavenging ability through the photothermal effect, while maintaining biocompatibility and degrading safely over time [143]. Although this system was designed for an ALI model, the underlying mechanism, ROS overproduction and inflammatory cytokine release, is extremely relevant to asthma pathogenesis [144]. Although PDA coated cerium dioxide nanozymes have not been tested specifically in asthma, they have shown good ability to scavenge intracellular ROS and reduce inflammatory responses in related lung injury, and this makes them great candidates for use in asthma therapy [143]. The similar overproduction of ROS and also immune dysregulation that happen in both ALI and asthma suggest that similar outcomes will be achieved in these asthma-specific models [143]. Overall, these results demonstrate the therapeutic potential of cerium oxide-based nanozymes in inflammatory airway diseases. Their intrinsic oxidative and hydrolase-like activities further contribute to modulating oxidative stress and inflammation, supporting their potential as multifunctional agents for future asthma research. Additionally, their unique redox cycling enables sustained antioxidant effects.

Also, Platinum-based nanozymes (PtNZs) reduce pulmonary inflammation by scavenging ROS through catalytic mechanisms that mimic the activities of CAT and SOD [145]. Their dose-dependent biocompatibility and immunocompatibility have been demonstrated across multiple cell types and tissues in both in vitro and in vivo studies [145]. Moreover, through precise control of surface engineering, size, and morphology, PtNZs can be directed toward specific inflammatory cell populations, thereby limiting cytokine production and modulating leukocyte-mediated inflammatory responses [146,147]. While evaluated mainly in ALI models, the nanozyme effects on ROS regulation and inflammatory signaling may hold relevance for asthma due to shared oxidative stress mechanisms. However, given the chronic and endotype-dependent nature of asthma, these results are considered mechanistic references requiring validation in asthma-specific models.

## 6. Nanozyme-Based Drug Delivery Systems for Asthma

### 6.1. Targeting Precision and Lung-Specific Delivery of Nanozymes

Delivering nanozymes to the airways and alveoli is an effective way to treat asthma with nanozymes [148], because inflammation is localized within the airways and alveoli. Inhalation is the most effective route to achieve high local concentration while minimizing systemic exposure [149], but this depends on the size of particles. If the particles are between 0.003 and 5 microns, they will be deposited by the sedimentation mechanism in the trachea, bronchioles, and alveoli region [150]. Dry powder inhalers, material-dose inhalers, and nebulization are examples of advanced inhalable devices that control pulmonary administration [151]. Surface modulation can also enhance the targeting of nanozymes, such as ligand–surface interaction, antibody conjugation, and PEGylation, which increases stability and reduces rapid immune clearance [152].

### 6.2. Smart and Stimuli-Responsive Nanozyme Carriers

Stimuli-responsive nanozymes are types that can adapt to environmental stimuli, allowing for selective release, regulation of catalytic activity, or synergistic therapy. This smart responsiveness significantly enhances the accuracy and outcomes of therapy [153]. Advanced stimuli-responsive nanozymes include those that respond to pH, glucose, other enzymes, light, ultrasound, and magnetic fields [153,154].

In asthma, the airways in the lungs become inflamed, becoming a local primary source of harmful ROS, such as hydrogen peroxide, which contributes to tissue damage and worsens disease symptoms [155]. To counter this, a novel strategy for creating nano systems based on ROS-responsive materials is gaining interest due to its potential to increase drug availability at the site of action, thereby improving therapeutic efficacy and minimizing systemic side effects, while also reducing the required dosage [156].

Accordingly, increasing research efforts have been directed towards the creation of ROS-sensitive chemical linkers such as thioketal, boronic ester, or thioether bonds, which remain stable in normal physiological conditions but degrade in the presence of elevated ROS levels, and this indicates their encouraging potential for precise diagnosis and successful treatment [157]. Numerous carrier systems for ROS-mediated drug delivery have been developed by the careful application of ROS-responsive functional moieties [158].

Overall, a potential approach to treating asthma is the use of nanozyme systems that combine catalytic antioxidant activity with redox-responsive medication release. These intelligent materials assist in regulating the immune response and enhance the accuracy of drug delivery, in addition to reacting to the oxidative environment of inflammatory lungs. However, further studies are needed to confirm safety, efficiency, and medical benefits in the clinical management of asthmatics.

### 6.3. Surface Functionalization Nanozymes

Surface functionalization is the process of altering a surface’s characteristics to provide it with physical, chemical, or biological features other than those it originally had. This procedure of enhancing surface properties has numerous uses, including in medical and material science [159]. It can be utilized to enhance the functionality of materials in a range of applications, for example, improving catalysts and enabling drug delivery systems. Functionalization may be utilized to give specific properties to a material, such as improved biocompatibility [160]. Overall, functionalization is an effective method for altering material properties, with the potential to transform a wide range of fields and industries [159]. In the context of asthma, where inflammation and oxidative stress demand targeted delivery to airway tissues, surface functionalization offers a platform to combine targeting ligands with antioxidant and anti-inflammatory activity.

One widely used strategy is coating the surface of NPs using PEG, often known as “PEGylation.” This technique reduces immunogenicity and protects the surface from aggregation, opsonization, and phagocytosis, extending systemic circulation time [161]. PEGylated nanozymes can increase the chance of reaching inflammatory airway sites in pulmonary applications by improving mucus penetration and prolonging residence time in the lungs [162].

Prussian Blue (PB) nanozymes, which are rich in surface functional groups such as carboxyl and hydroxyl groups, offer a versatile platform for functionalization to enhance systemic circulation and lesion-targeting abilities [163,164]. PEG coatings on PB nanozymes are frequently utilized to prolong blood circulation time, and targeting ligands like Arginine–Glycine–Aspartate peptide (RGD), as well as antibody fragments, can be added for improved lesion specificity [165,166]. Additionally, PB’s porous structure facilitates the effective encapsulation and regulated release of therapeutic medications, such as corticosteroids, siRNAs, and small-molecule antioxidants, allowing for multifunctional therapy in the complex inflammatory pathogenesis of asthma [165].

Multifunctional designs further expand therapeutic potential. For example, Prussian Blue Nanozyme Functionalized with P-selectin Peptides and Mannose (PBNZ@PP-Man), a dual-ligand PB-based nanozyme, combines intrinsic enzyme-mimetic catalytic properties with targeted delivery of drugs and immunomodulatory benefits [167]. To boost the specificity of targeting, the nanozyme surface was modified using two ligands, which are P-selectin-binding peptides (PP) along with mannose. The PP component particularly targeted active endothelial cells, which overexpressed P-selectin; however, mannose increased macrophage-specific uptake as well as apoptotic cell clearance [167]. Although this was initially used in vascular inflammation, a similar strategy can be used in asthma by inhibiting the overexpressed adhesion molecules in airway endothelium and pro-inflammatory macrophages in the lung tissue. Such constructs would be able to treat both oxidative stress and immune dysregulation in asthma by combining intrinsic antioxidant activity with site-specific delivery.

Overall, the integration of coatings, ligand conjugation, and other elements in nanozyme surface functionalization gives us a versatile and powerful approach to enhance therapeutic delivery, improve biocompatibility, and maximize catalytic performance in airway inflammatory diseases. This technique has the potential to greatly enhance asthma treatment outcomes by addressing both the biochemical and cellular causes of illness.

### 6.4. Co-Delivery Nanozymes

Nano methods of delivery have greatly contributed to the treatment of numerous conditions. However, monotherapy, which delivers only one medicine, is insufficient to treat many diseases. The nano-based co-delivery method emerged as a result of successfully addressing issues of monotherapy [168,169]. First, the combined delivery formulation can accurately transport the loaded drug into the intended cells while lowering systemic toxicity through targeting inflamed areas by modifying the surface with particular moieties. Meanwhile, it slows the release process by encapsulation of the medication, thus enabling a relatively large dosage distribution while minimizing negative effects [169,170]. Co-administration of drugs improves therapeutic efficacy and circumvents pharmacological barriers in a number of ways [169].

## 7. Green Synthesis and Biocompatibility of Nanozymes in Asthma

Conventional methods of synthesizing nanozymes using metals and metal oxides have demonstrated adverse health effects. Researchers have directed their efforts towards green synthesis in order to increase biocompatibility and reduce toxicity [171,172,173].

**Figure 4:**
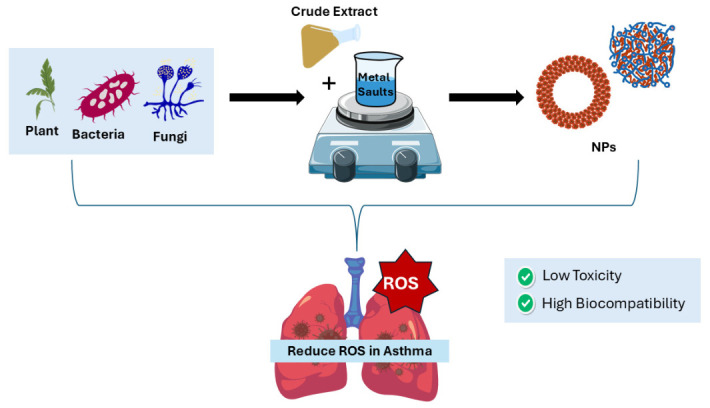
Green synthesis of NPs for asthma therapy.

As illustrated in Figure 4. Green synthesis of NPs is a process that employs biological organisms as part of the synthetic process, typically using plants, fungi, bacteria and algae. This approach decreases the usage of hazardous chemicals, lowers energy consumption, and increases the biocompatibility of the nanozymes, making them more suitable for biomedical applications such as asthma therapy [174]. As a result, researchers are currently searching for a green blend method to exclude toxic synthetics during NP manufacturing [174,175]. Overall, nanozymes are biocompatible in vivo and in vitro. However, current safety research focuses on just a few key toxicological endpoints, making it hard to undertake a thorough and comprehensive analysis. As a result, various constraints must be addressed in order to ensure safe therapeutic usage [176]. Although nanozymes work side by side with natural antioxidant enzymes to eliminate ROS in asthma, and despite their many advantages, such as tunable size, stability, and the ability to mimic natural enzyme activity, they still face some intrinsic limitations [174,177]. Several studies have reported that some nanozymes are cytotoxic because they accumulate in lung tissue and their strong interactions with lipids, proteins, carbohydrates, and DNA, which may impair cell membrane integrity and disrupt enzymatic processes [174,177]. To effectively treat asthma, further studies are required to improve the in vivo behavior, biocompatibility, targeting, and catalytic activity of green synthesis nanozymes.

## 8. Personalized Nanozyme Therapy for Asthma Therapy

Personalized medicine is a new and promising field of medicine that examines each patient’s unique genetic makeup, including specific biomarkers, to make decisions about how to prevent, diagnose, and treat diseases [178,179]. Asthma is a promising model for personalized treatment due to its complex nature caused by genetic, environmental, and epigenetic variables [180]. Also, personalized medicine involves categorizing asthma based on endotypes, which link observable traits to distinct immune systems. Identifying these endotypic mechanisms is critical for precisely assessing patients and designing therapeutic approaches employing new biological agents that target specific immune pathways [181]. A biomarker is any objectively measured characteristic used to indicate disease diagnosis, to predict the response to treatment, and to monitor disease progression. To be applicable in clinical practice, a biomarker should be able to define a phenotype and/or an endotype, predict and evaluate treatment response, and monitor disease progression [178,182,183]. As we enter the era of personalized medicine, identifying the perfect biomarker with the above-mentioned characteristics is urgently needed, especially in allergic and other T2-overlapping severe asthma endotypes, for which the choice of the ideal treatment for each patient is quite challenging [178]. Asthma is caused by a pathogenesis involving the innate and adaptive immune systems and epithelial cells, which leads to mucus overproduction, airway remodeling, and bronchial hyperreactivity [184]. It was traditionally thought to be a Th2-driven allergic disease [185]. However, further research showed the existence of other phenotypes and endotypes such as eosinophilic, neutrophilic (Th17-related), and mixed types, each with distinct underlying immunological processes [179,186]. Recent research has highlighted the crucial role of innate lymphoid cells (ILC-2), which produce Th2-type cytokines (IL-4, IL-5, and IL-13) that promote airway inflammation even in non-atopic individuals. Specifically, these cells react to “alarmins” produced from epithelial cells, including TSLP, IL-25, and IL-33 [179,187]. TSLP increases corticosteroid resistance and adaptive immune activation, IL-25 and IL-33 promote T2 inflammation through nuclear factor kappa-light-chain-enhancer of activated B cells (NF-kB) signaling and suppression of tumorigenicity 2 (ST2) receptor pathways [8,187,188]. This complicated immunological interaction explains why some individuals respond poorly to traditional treatments [8,187]. Consequently, understanding the molecular and immunological heterogeneity of asthma is crucial for enhancing the compatibility and reducing the toxicity of nanozymes, thereby minimizing side effects and advancing the goal of successfully applying personalized medicine to asthmatics.

## 9. Biosafety Assessment of Nanozymes

Biosafety evaluation is an essential prerequisite for nanozyme-based therapeutics. It includes rigorous in vitro and in vivo investigations to exclude potential adverse effects on healthy tissues. Key benchmarks for assessing this include cell viability, hemocompatibility, oxidative stress, cell death pathways, inflammatory responses, mitochondrial function, and systemic toxicity [174,189]. Multiple studies have reported the therapeutic efficacy of tested nanozymes in disease models without evidence of acute systemic toxicity, supporting the feasibility of inhalation-based nanozyme therapies under controlled conditions [107]. In vitro evaluations demonstrated that the nanozymes effectively protected macrophages from oxidative stress without inducing detectable cytotoxicity, indicating high cellular biocompatibility [170]. However, long-term in vivo toxicity, biodistribution persistence, and immunogenicity were not comprehensively investigated [170]. Another study indicates prolonged residence in the lungs may result in chronic inflammation, pulmonary fibrosis, or granuloma formation [190]. Also, to enhance biosafety, advanced targeted delivery strategies are being engineered, including cell membrane-coated NPs (e.g., neutrophil-mimetic systems), to promote selective accumulation of nanozymes within inflamed pulmonary tissues while minimizing off-target toxicity [191]. Accordingly, further systematic studies are required to fully elucidate their safety and translational potential for clinical applications [170]. Although nanozymes have demonstrated favorable pulmonary biocompatibility in models of acute lung injury, their biosafety in asthma has not been sufficiently characterized. Asthma presents distinct pathological features, including chronic airway inflammation, eosinophilic infiltration, mucus hypersecretion, and airway remodeling, which may influence nanozyme deposition, clearance, and immunological interactions.

## 10. Challenges and Limitations

Nanozymes hold therapeutic promise for the treatment of asthmatics, yet their clinical translation is limited by several significant challenges. Current toxicological assessments remain incomplete, as most studies evaluate only a narrow range of safety endpoints, preventing a full understanding of long-term risks, biodistribution, and in vivo behavior [192]. Despite the apparent benefits of nanozymes, whether chemical or green synthesis, there are limitations and challenges for asthma treatment. One of the limitations of chemically synthesized nanozymes is that they may show cytotoxicity for several reasons. They can induce ROS, which can worsen asthma inflammation. They also have weak targeting, low catalytic activity, poor substrate affinity, and a risk of accumulation in organs such as the lungs and others [174,176]. On the other hand, although green-synthesized nanomaterials are generally more biocompatible and safer, they still face limitations, such as poor control over particle size and morphology, and they have lower catalytic activity [193]. Both approaches face challenges in targeted delivery to inflamed airway tissues, immune recognition, weak substrate specificity, stability in the oxidative environment of asthmatic lungs and incomplete in vivo safety evaluation [174,176]. Overcoming these limitations is important for developing nanozymes with improved targeting, controlled biodegradability, and enhanced biocompatibility, which is essential for successful clinical translation in asthma treatment [194].

Although nanozymes possess desirable characteristics such as tunable size, stability, and the ability to mimic natural antioxidant enzymes, they still exhibit intrinsic drawbacks that restrict their therapeutic efficiency [174]. Primary concerns are the potential cytotoxicity and biocompatibility issues associated with certain nanozymes, which may accumulate in lung tissue or interact adversely with cellular components, including lipids, proteins, carbohydrates, and DNA, leading to disruption of membrane integrity and interference with essential biochemical pathways [195]. Moreover, their in vivo performance, including biodistribution, immunological compatibility, targeting specificity, and catalytic activity, remains insufficiently optimized for the complex environment of inflammatory asthmatic airways where oxidative stress and immune dysregulation coexist [196]. Despite their promising therapeutic potential, the application of nanozymes in asthma remains insufficiently investigated, with most existing studies focusing on general lung injury or inflammatory models rather than asthma specifically. Therefore, further asthma-specific preclinical and clinical studies are essential to fully evaluate their therapeutic efficacy, targeting capability, and long-term safety. To ensure safe and effective applications, future research must concentrate on optimizing nanozyme design, improving biocompatibility, preventing tissue accumulation, and expanding toxicological studies to completely define both short-term and long-term biological effects.

## 11. Conclusions

Nanozymes are potentially important for current and future asthma treatments. They have notable immunomodulatory and redox-responsive features, making them a prospective therapy option for asthmatics. As nanozymes mimic natural antioxidant enzymes, they can scavenge excess ROS in an efficient way; they can also restore the redox balance by protecting airway epithelial cells and diminishing chronic inflammation. Their broad-spectrum functions enable catalytic antioxidant action and targeted drug delivery, overcoming the limitations of conventional asthma treatments. Due to developments in surface-functionalized and stimuli-responsive nanozymes, there have been enhancements in biocompatibility with lung tissues, allowing for regulated drug release in redox-active environments and improvements in therapeutic precision. Furthermore, eco-friendly, low-toxicity nanozymes that are appropriate for biomedical applications have been shown to be suitable for green synthesis techniques. The development of co-delivery systems that combine nanozyme catalysis and pharmaceutical drugs also offers potential treatments that reduce negative drug effects and increase efficiency. Despite these significant research gains, more studies are still needed to completely understand the in vivo behavior, biodistribution, and long-term safety of nanozymes. Future studies will focus on improving the design of nanozymes, assessing optimized uses according to asthma endotypes, and performing clinical trials to validate their therapeutic capabilities. Nanozyme-based treatments, which combine nanotechnology, enzymatic catalysis, and precision medicine, have enormous potential to transform asthma care and facilitate the development of safer, more efficient, and customized treatment approaches.

## Figures and Tables

**Table 1:** Nanozyme-based therapeutic platforms evaluated respiratory inflammatory disease models such as ALI, highlighting physicochemical properties and therapeutic outcomes relevant to asthma pathophysiology.

Nanozyme	Particle Size	Morphology	Zeta Potential	Effects	Model	Target Disease	Ref.
P@Co	59.9 ± 9.4 nm	Spherical	−44.5 ± 0.4 mV	ROS Scavenging Anti-inflammatory effect M2 Polarization NIR Enhancement	In Vitro and In Vivo	ALI	[106]
CeO_2_-NZs	25 nm	Ultrasmall nanodots	-	ROS scavenging Cyclic regeneration	In Vitro and In Vivo	ALI	[107]
CDs	<10 nm	Spherical	-	ROS scavenging Oxidative stress attenuation in lung tissues	In Vitro and In Vivo	ALI	[108]
VTMn-CD	2.25 ± 0.42 nm	Spherical	−15.5 ± 0.9 mV	ROS Scavenging Anti-inflammatory effect	In Vitro and In Vivo	ALI	[109]

## Data Availability

No new data were created or analyzed in this study. Data sharing is not applicable to this article.

## Abbreviations

AHR	Airway Hyperresponsiveness
ALI	Acute Lung Injury
CAT	Catalase
CAT-OXD	Catalase–Oxidase
C-dots	Carbon Dots
CCL5	C-C Motif Chemokine Ligand 5
CeO_2_	Cerium Dioxide
Ce@P	Polydopamine-Coated Cerium Dioxide Nanozyme
COX-POD	Cholesterol Oxidase–Peroxidase
DAMPs	Damage-Associated Molecular Patterns
DCs	Dendritic Cells
ECP	Eosinophil Cationic Protein
GSH	Glutathione
H_2_O_2_	Hydrogen Peroxide
HDM	House Dust Mite
HO	Hydroxyl Radical
HRP	Horseradish Peroxidase
IFNGR	Interferon Gamma Receptor
IgE	Immunoglobulin E
LTB4	Leukotriene B4
M1	Classically Activated Macrophages
M2	Alternatively Activated Macrophages
MBP	Major Basic Protein
MOF	Metal–Organic Framework
NAA	Non-Allergic Asthma
NOX	NADPH Oxidase
NOS	Nitric Oxide Synthase
NLRP3	NOD-like receptor family pyrin domain-containing 3
NPs	Nanoparticles
O_2_**^.^**^−^	Superoxide Anion
OXD	Oxidase
PBNZ@PP-Man	Prussian Blue Nanozyme Functionalized with P-
PtNZs	Platinum-based nanozymes
PDA	Polydopamine
POD	Peroxidase
PASMCs	Pulmonary Artery Smooth Muscle Cells
PAF	Platelet activation factor
PGD2	Prostaglandin D2
ROS	Reactive Oxygen Species
SOD	Superoxide Dismutase
ST2	Suppression of Tumorigenicity 2 Receptor
Th2	T Helper Type 2
TNFR1	Tumor Necrosis Factor Receptor 1
TNFR2	Tumor Necrosis Factor Receptor 2
TSLP	Thymic Stromal Lymphopoietin
WHO	World Health Organization
ASM	Airway Smooth Muscle
LPS	Lipopolysaccharide
Cys-LTs	Cysteinyl Leukotrienes
SAzyme	Single-Atom Nanozyme
PS-PEG	Polystyrene–Polyethylene Glycol
AuNPs	Gold NPs
M-CAT@NGs	Catalase-Loaded Nanogels
MAPK	Mitogen-Activated Protein Kinase
MHC	Major Histocompatibility Complex
HDAC2	Histone Deacetylase 2
BG	β-Glucan
NRF2	Nuclear Factor Erythroid 2-Related Factor 2
GPx	Glutathione Peroxidase
PEG	Polyethylene Glycol
NF-Κb	Nuclear factor kappa-light-chain-enhancer of activated B cells
TRX	Thioredoxin
TXNIP	Thioredoxin-interacting protein
PP	P-selectin-binding peptides
ARDS	Acute respiratory distress syndrome
CuS NPs	Copper sulfide NPs
GDYO	Graphdiyne oxide
